# Mosquitoes Used to Draw Blood for Arbovirus Viremia Determinations in Small Vertebrates

**DOI:** 10.1371/journal.pone.0099342

**Published:** 2014-06-05

**Authors:** Rebekah C. Kading, Brad J. Biggerstaff, Ginger Young, Nicholas Komar

**Affiliations:** Division of Vector-Borne Diseases, Centers for Disease Control and Prevention, Fort Collins, Colorado, United States of America; Virginia Tech, United States of America

## Abstract

Serial samples from the same individuals may be required for certain virological studies, however, some small animals cannot easily be blood-sampled. Therefore, we evaluated the use of *Culex quinquefasciatus* Say and *Aedes albopictus* Skuse mosquitoes as “biological syringes” to draw blood for virus titer determinations in small vertebrates. Groups of chicks (*Gallus gallus*), hamsters (*Mesocricetus auratus*), and house sparrows (*Passer domesticus*) were experimentally infected with West Nile virus (WNV) or Highlands J virus (HJV). In general, good correlation was seen between mosquito- and syringe-derived blood samples at titers ≥5.0 log_10_ pfu/mL serum as compared with titers <5.0 log_10_ pfu/mL serum for chicks, hamsters, and sparrows. Ninety-two percent (24/26) of sparrows with virus titers >10^5^ pfu/mL serum had mosquito- and syringe-derived titers within one log of each other. Sparrow viremia profiles generated from single mosquito blood meals and syringe were not significantly different (p>0.05). This technique is valuable for assessing the roles of small vertebrates in the ecologies of arboviruses, and could be used in applications beyond virology and infectious diseases, when <10 µL of whole blood is required.

## Introduction

The use of animals in laboratory studies is paramount to understanding biological, ecological, and clinical components of arboviral infection. Experimental infection studies have provided valuable information on viral factors such as virulence and pathogenesis, and on host factors such as susceptibility, reservoir competence and immune response. Integral to all of these types of studies is the ability to obtain blood samples from animals in a manner that both meets the specific aims of the research and minimizes stress to the animals.

Many vertebrate infection experiments require repeated blood sampling of the same individuals over specific time intervals. However, some animals such as mice, and small birds and reptiles, cannot easily be bled repeatedly due to their small size and limited blood volume or lack of sufficient vein presentation. Different groups of animals are often bled on alternating days due to limitations in the amount of blood that can safely be taken from an individual animal over a given time period [1]–[4]. Animal care guidelines restrict blood acquisition from experimental animals to 10% of blood volume (roughly 1% of body weight) within a 3–4-week period, or 1% blood volume for repeated bleeds at shorter intervals (once per 24-hours) [5]. Traditional methods of blood collection requiring needles or lancets typically can control loss of blood to within 0.1 mL, which permits daily sampling for one week in vertebrates weighing approximately 70 g and larger. A technique is needed to sample 0.01 mL or less daily without risk of blood-loss due to accidental hemorrhage. Such a technique would be useful in determining the viremia profile, or daily virus titer in the peripheral blood following exposure to a virus, in experimentally-infected animals which would otherwise be difficult or impossible to measure.

Yuill [6] reported a proof of principle experiment in which mosquitoes were used as tools to draw blood from viremic hosts, but the technique was not compared to standard methods and viremia titers were not measured. Mahmood et al. [7] measured the daily virus titer in five nestling mourning doves infected with St. Louis encephalitis virus using both jugular venipuncture as well as groups of mosquitoes. Analysis of virus titers in mosquito blood meals following engorgement demonstrated the infectiousness of 1–5-day old nestling doves to mosquitoes and approximated a viremia profile, supporting the potential role of nestling doves as virus amplifying hosts. However, direct comparisons between mosquito- and syringe-derived blood samples from individual birds were not rigorously evaluated in the context of using mosquito-derived samples as a proxy for syringe sampling. If mosquito-derived blood functions as syringe-derived blood for viremia determination, then very small vertebrates can be subject to experimental evaluation. Accordingly, the specific aims of this study were to 1) compare viremia titers derived from blood drawn simultaneously by both methods (needle-syringe vs mosquito) and 2) compare viremia profiles obtained by each blood-collection method. For aim 1), we assessed two classes of vertebrate hosts, mammalian (hamster) and avian (chicken and house sparrow), using two different families of viruses, *Flaviviridae* (West Nile virus, WNV) and *Togaviridae* (Highlands J virus, HJV). For aim 2), we evaluated WNV and HJV in house sparrows.

## Materials and Methods

### Ethics Statement

All animal work was carried out in strict accordance with the recommendations in the Guide for the Care and Use of Laboratory Animals of the National Institutes of Health. The protocol was approved by the CDC Institutional Animal Care and Use Committee, Protocol # 07–011. All efforts were made to minimize suffering.

### Animals and Animal Care

Five to seven-day-old and two- to three week-old chicks (*Gallus gallus*) (Northern Colorado Feeder Company, Fort Collins, CO) and eight- to twelve-week-old female Syrian golden hamsters (*Mesocricetus auratus*) (Harlan Sprague Dawley Inc., Indianapolis, IN) were used. Chicks were housed individually and hamsters in groups of two in large mouse cages. House sparrows (*Passer domesticus*) were captured by mist nets (Avinet Inc., Dryden, NY) in Weld County, Colorado and transported in rubber-coated wire-mesh finch flight cages (PetCo Inc., Fort Collins, CO). Sparrows were aged using standard plumage characteristics [8], sexed, and banded with uniquely-numbered aluminum leg bands. A 0.3-mL blood sample was taken by jugular venipuncture from each bird. Blood was collected directly into Microtainer serum separators (Beckton-Dickinson Co., Franklin Lakes, NJ). Sparrows were given a two-week acclimation period in captivity prior to experimentation. During this period they were physically examined by the attending veterinarian, treated with anti-helminth medication (Avermectin), and transferred in groups of 12 to screened non-human primate cages equipped with perches. Animals were provided food and water *ad libidum*: standard rodent feeder diet (hamsters), hen grower pellets (chicks), or mixed bird seed (sparrows). Wild-caught sparrows were confirmed negative for neutralizing antibody to WNV and HJV by plaque reduction neutralization test (PRNT) [9], using challenge doses of approximately 100 plaque forming units (pfu) of WNV (strain NY99-4132) or HJV (strain B230). Birds with >70% neutralization of WNV were excluded from experimental infection with that virus. Conservatively, birds with >50% neutralization of HJV were excluded from infection with that virus due to the probability that birds exhibiting alphavirus neutralizing antibody most likely had a past infection with a member of the Western equine encephalitis virus (WEEV) complex.

Prior to blood sampling, hamsters were anesthetized with 0.025 mL/10 g body weight using a mixture of ketamine (dose 200 mg/kg) and xylazine (10 mg/kg), injected intraperitoneally (ip). Chicks and sparrows were administered 0.01 mL/10 g body weight ketamine (dose 50 mg/kg) and xylazine (10 mg/kg) mixture intramuscularly (im). Following experimentation, animals were euthanized by cervical dislocation performed under anesthesia.

### Mosquitoes

*Aedes albopictus* (Lake Charles, LA strain) eggs were hatched by placing egg papers submerged in de-ionized water in a vacuum for approximately 10 minutes. Flooded eggs were left in the vacuum for one hour before transferring to larval rearing pans containing 0.4% liver powder solution. *Culex quinquefasciatus* (Sebring strain) larvae were reared on liver powder solution and rabbit chow. Adult mosquitoes were maintained in environmental chambers held at 27°C with approximately 90% humidity, on a 12L:12D cycle. Mosquitoes were provided 5% sucrose solution which was removed 24–48 h prior to experimental blood feeds. Adult mosquitoes were at least four days old at the time of experimentation.

### Viruses and Virus Inoculation

Animals, viruses, and sampling days are presented in Table 1. Viruses were selected for their viremogenic potential in chicken, hamster, and house sparrow [1], [10]–[13]. All viruses were obtained from the reference collection at the Division of Vector-Borne Diseases, Centers for Disease Control and Prevention (Fort Collins, CO). Animals were needle-inoculated subcutaneously with approximately 10^4^ pfu of WNV (strain NY99-4132) or HJV (strain B230) virus suspension in 0.1 mL BA-1 media (Hanks M-199 salts, 0.05M Tris pH 7.6, 1% bovine serum albumin, 0.35 g/L sodium bicarbonate, 100 U/mL streptomycin, 1 µg/mL Fungizone). All birds were inoculated in the breast and hamsters in the lower abdomen with a 26 g ½-in needle attached to a tuberculin syringe. Inoculations were accompanied by the bite of 1–3 uninfected *Ae. albopictus* mosquitoes at the site of virus inoculation to more closely simulate natural infection [4].

**Table 1:** Animals, viruses, and days post inoculation each animal was sampled by syringe and mosquito.

Animal	N	HJV	WNV	Days post
		Inoculum	inoculum	inoculation blood samples were taken
Chick (5–7 days old)	HJV:0; WNV:4	10^4.0^ pfu+mosquito saliva	10^4.0^ pfu+mosquito saliva	HJV: Days 1 and 2; WNV: Days 2 and 3
Chick (2–3 weeks old)	HJV:10; WNV:10	10^4.3^ pfu+mosquito saliva	10^4.0^ pfu+mosquito saliva	HJV: Days 1 and 2; WNV: Days 2 and 3
Hamster	HJV:10; WNV:10	10^4.0^ pfu+mosquito saliva	10^4.0^ pfu+mosquito saliva	HJV: Days 1–3; WNV: Days 3 and 4
House sparrow	HJV:24; WNV:36	10^4.6^ pfu	10^2.6^ pfu	HJV: Days 1–3; WNV: Days 1–4

Virus inocula are given as pfu per 0.1 mL BA-1 media.

### Blood Sampling

Between days one and four post-inoculation when peak viremia was expected [1], [13]–[14], blood was collected by mosquitoes and syringe. *Ae. albopictus* and *Cx. quinquefasciatus* mosquitoes were starved 24–48 h prior to exposure to infected animals, and fed either by placing anesthetized animals onto screened pint cups containing 10–15 female mosquitoes or by holding a 2-dram screened vial containing 5–10 female mosquitoes to a manually-restrained animal (two- to three-week-old chicks only). After feeding, mosquitoes were killed by freezing. Fully engorged mosquitoes were homogenized in TenBroeck glass tissue grinders containing either one or two milliliters of BA-1+20% fetal bovine serum (FBS), individually or in pools of two to five mosquitoes. Mosquito homogenates were transferred to 2.0 mL microcentrifuge tubes and centrifuged for two minutes at approximately 6,000×g. Supernatants were transferred to cryovials and stored at −80°C until testing (chicken and hamster). House sparrow samples were immediately inoculated onto Vero (African green monkey kidney) cell monolayers.

Syringe samples were taken within 20 minutes of mosquitoes feeding on each animal. 0.1 mL blood was collected using a syringe attached to a 26 g ½-in SubQ needle. Blood was collected by jugular venipuncture of chicks on two occasions (one day apart) for each chick, and of sparrows on one or two occasions (three days apart), and by cardiac puncture of hamsters (post-mortem). Syringe samples were either expelled directly into Microtainer serum separators and centrifuged to isolate the serum for testing, or diluted in 450 µl BA-1 diluent (to achieve a 1∶10 dilution of serum). Blood samples were allowed to coagulate at room temperature for up to 30 minutes before placing them on ice.

### Testing Methods

Blood samples taken by syringe and mosquito were titrated simultaneously using the double-overlay Vero cell plaque assay [9]. Vero cell monolayers in 6-well polystyrene culture plates (Costar Inc, Cambridge, MA) were inoculated with 0.1 mL of diluted serum in duplicate. For syringe samples, 10-fold dilutions from 10^−1^ to 10^−9^ were tested, and for homogenized mosquito samples, 10-fold dilutions from undilute to10^−5^ were tested. The second overlay, containing neutral red, was added on the second day post-inoculation (dpi) for HJV, and the third dpi for WNV. HJV plaques were counted at 2 and 3 dpi, and WNV at 3 and 4 dpi to ensure no plaques were missed. For titer calculations, 2-µl blood meals (1 µl serum) were assumed [15]–[17]. Mean daily titers were calculated by averaging the log titers for multiple mosquitoes fed on the same animal, then averaging the log titers for animals each day.

### Test for Virus Adsorption to Mosquito Tissues

To ensure that any differences in titer between mosquito and syringe samples were not due to virus binding to mosquito tissues during blood feeding or grinding, six replicates each of 1 mL BA1+20% FBS containing approximately 10^2^ pfu HJV or WNV were incubated at 28°C for 45 minutes with or without a homogenized mosquito. Virus titers of paired samples were compared by Vero cell plaque assay.

### Statistical Analysis

Three questions of interest concerning the association between virus log titer measurements obtained via syringe sampling and mosquito sampling of blood from a given animal were addressed: 1) as a function of the syringe-sampled log titer, what is the probability that the mosquito-sampled log titer would be positive, i.e., would detect virus? 2) among animals with positive log titer measurements using both mosquito and syringe sampling, at what syringe-sampled log titer would there be a 25%, 50%, or 75% probability that the corresponding mosquito-sampled log titer would be measurable, and what is the expected mosquito-sampled log titer as a function of the syringe-sampled log titer? 3) for each of the questions 1 and 2, do these associations vary by animal host (chicken, hamster, sparrow) or virus (HJV, WNV)?

A mixed discrete and continuous regression model was used to evaluate all three of these questions simultaneously. The discrete component of the model consisted of a logistic regression for log titer positivity, where the logit of the probability of a positive mosquito-measured log titer was modeled as a linear function of the syringe-measured log titer. The continuous component of the model was a linear model of the mosquito-measured log titer as a function of syringe-measured log titer, though no intercept was included in the model to reflect the fact that no mosquito-sampled values would be positive when the syringe-sampled values were 0. For both discrete and continuous components of the model, variables were included to permit different intercepts (discrete only) and slopes for animal and virus, including interactions. The models were fit using maximum likelihood, 95% profile confidence intervals (CI) for the model parameters were computed, and models were compared and selected using the likelihood ratio test and 5% significance. Inversion of the logistic model component provided estimates and 95% CIs of syringe-measured log titer values expected to yield positive mosquito-measured log titer values with probabilities of 25%, 50%, 75% and 90%. Standard regression diagnostics were used to evaluate model assumptions.

Analyses were carried out and graphs produced in the R statistical software package [18], and maximum likelihood (ML) estimation and associated inference was done using the bbmle package in R [19].

For analysis of WNV and HJV viremia profiles (log titer over time), we took two approaches. For a direct comparison of mosquito-sampled versus syringe-sampled log ‘titers at each dpi separately, we used the paired t-test, which accounts for the paired observations on individual house sparrows. These comparisons by dpi within virus were adjusted for multiple comparisons using the Bonferroni adjustment. We then used mixed linear models to evaluate potential trends in the association between dpi and titer, including collection type (mosquito or syringe) as predictors; interaction of collection type with dpi was also included. Because titers were only measured at four time points, we interpret results of this modeling as indicative of general trends in the titers over time, rather than as definitive descriptions of the profiles. Estimation was done using ML when comparing fixed effect model parameters using the likelihood ratio tests, while final model parameters were estimated with restricted ML (REML). For these analyses, there were minimal 0-valued titers, so we did not model the probability of obtaining a 0 titer as a separate model component. Models were fit in R using the nlme package [20].

## Results

### Mosquitoes

Starved *Ae. albopictus* mosquitoes usually fed within a few minutes of exposure to the animal, whereas *Cx. quinquefasciatus* did not feed as readily. Therefore, feedings with *Cx. quinquefasciatus* were stopped after the first few days and continued with only *Ae. albopictus*. *Aedes* mosquitoes fed to repletion more quickly when starved 48 h and fed on anesthetized animals as compared with manually-restrained animals.

### House Sparrow

All birds were >3 months old at the time of capture. Initial sampling indicated that 18% (13/71) of sparrows were positive for neutralizing antibodies to WNV, and 11% (8/71) were positive for alphavirus-neutralizing antibodies. Birds with prior exposure to WNV were excluded from experimental infection with WNV. Similarly, birds with alphavirus neutralizing antibodies were excluded from experimental infection with HJV.

### Regression Modeling

The mixed discrete and continuous regression model fits and model comparisons using the likelihood ratio test resulted in a final model with no statistically significant differences in the logistic component’s intercept and slope by either animal or virus. In contrast, a statistically significant difference was found in the slopes of the linear component by virus, though not by animal (Table 2). Model parameter estimates and 95% CIs for the final model are given in Table 3.

**Table 2:** Models fit and nested model comparisons made using the likelihood ratio test (chi-squared test, df = degrees of freedom).

Model	Logistic	Linear	Comparator Model	Total df	Deviance	Chi-sq	df	p-value
1	AVi	AVi	–	19	221.4			
2	AV	AVi	1	14	223.3	1.9	4	0.76
3	AVi	AV	1	17	225.0	3.6	2	0.16
			1	13	226.9	5.5	6	0.48
4	AV	AV	2			3.6	2	0.16
			3			1.9	4	0.76
5	AV	A	4	12	231.4	4.5	1	**0.03**
6	AV	V	4	11	229.8	2.9	2	0.23
7	A	AV	4	11	230.0	3.1	2	0.21
8	V	AV	4	9	232.9	6.0	4	0.20
9	A	A	5	10	234.5	3.1	2	0.21
			7			4.5	1	**0.03**
10	A	V	6	9	233.0	3.1	2	0.21
			7			2.9	2	0.23
11	V	V	6	7	235.8	6.0	4	0.20
			8			2.9	2	0.23
12	V	A	5	8	237.4	6.0	4	0.20
			8			4.5	1	**0.03**
13	A	1	9	8	239.5	5.0	2	0.08
			10			6.5	1	**0.01**
14	1	A	9	6	239.1	4.6	4	0.33
			12			1.8	2	0.42
15	V	1	12	6	242.3	5.0	2	0.08
			11			6.5	1	**0.01**
16	1	V	10	5	237.6	4.6	4	0.33
			11			1.8	2	0.42
			13	4	244.1	4.6	4	0.33
			14			5.0	2	0.08
17	1	1	15			1.8	2	0.42
			16			6.5	1	**0.01**

Models are specified by their logistic and linear components, with “A” indicating a term for animal was in the model component, “V” indicating a term for virus was in the model component, and “I” the interaction between animal and virus was in the model component. For convenience, model labels are given in the first column (Model), and the nested/reduced model being compared to that is given as the Comparator Model, referenced by the Model label of column one. P-values <0.05 are in boldface.

**Table 3:** Parameter estimates and 95% confidence intervals for the final model relating mosquito-measured and syringe-measured titers.

Logistic component	Estimate	95% CI
Intercept	−4.37	−6.16, −2.94
Slope	1.20	0.83, 1.67
**Linear component**		
Slope – HJ	1.03	0.97, 1.09
Slope – WNV	0.93	0.89, 0.97
Variance	0.59	0.42, 0.85

Recall there was no intercept included in the linear component of the model.

### Probability of Detecting Virus in A Mosquito Blood Meal

**Figure 1:**
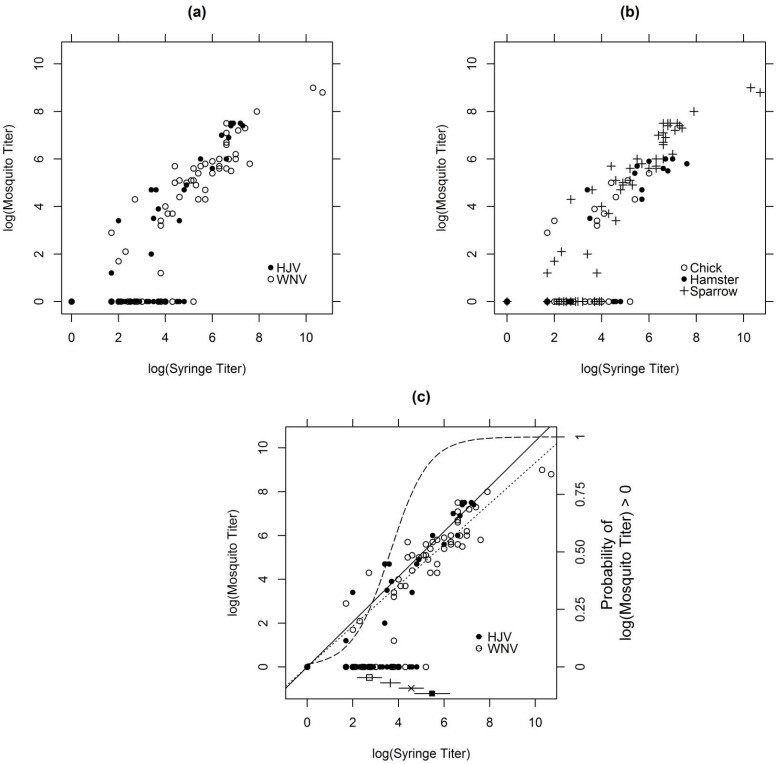
Corresponding virus titers (log_10_ pfu/mL serum) derived from mosquito- and syringe-drawn blood. Data for viruses (a) or animals (b). Panel (c) shows the data by virus with graphical representations of the linear (scale on left margin) and logistic (scale on right margin) components of the final fitted model. Also shown below the horizontal 0 in panel (c) are estimates and 95% confidence intervals for the syringe-measured titers that have 25% (o), 50% (+), 75% (×), and 90% (▪) probabilities of obtaining a positive mosquito-measured titer.

Chicken, hamster, and house sparrow all developed detectable viremia for both WNV and HJV infections. The probability of detecting virus in a mosquito blood meal increased with virus titer (as syringe-measured; Fig. 1). Detection of virus in a single mosquito blood meal was limited to titers >10^3^ pfu/mL serum, (approximately one pfu in one microliter of serum in a blood meal) because of volumetric constraints of the mosquito blood meal size. However, this obstacle was overcome by testing multiple individual mosquitoes fed on the same viremic animal. For a 25% probability of detecting virus in a single mosquito blood meal, the syringe log titer needed to be ≥2.72 log_10_ pfu/mL (95% CI 2.19–3.27), while for a 50% probability of detection, the syringe log titer needed to be ≥3.64 log_10_ pfu/mL (95% CI 3.20–4.08). Corresponding syringe log titers for 75% and 90% probabilities of detection were ≥4.56 log_10_ pfu/mL (95% CI 4.02–5.10) and ≥5.48 log_10_ pfu/mL (95%CI 4.71–6.24), respectively. These reference log titers are shown in Figure 1, panel (c).

### Accuracy of Mosquito Blood Meal Titers

In general, good correlation was seen between mosquito and syringe samples at titers ≥5.0 log_10_ pfu/mL serum as compared with titers <5.0 log_10_ pfu/mL serum (Fig. 1). Both estimates for the slopes by virus in the linear component of the regression model were essentially 1, indicating that when the virus was detected via mosquito sampling, the log titer results matched the syringe-measured results (Table 3). The mean difference in log titer between mosquitoes and syringe was not significantly different by virus or by day post inoculation, except for HJV 2 dpi(Table 4). Ninety-two percent (24/26) of sparrows with virus titers >10^5^ pfu/mL serum had mosquito- and syringe-derived titers within one log of each other. Of the remaining two birds with virus titers >10^5^ pfu/mL, the average mosquito titer was 1.1 log_10_ pfu/mL (SE+0.06) greater (HJV 1 dpi), and 1.8 log_10_ pfu/mL (SE±0.1) less (WNV 3 dpi) than the corresponding syringe titers, but the mean difference between mosquito- and syringe-derived titers at these time points across all birds and mosquitoes was not significant (Table 4).

**Table 4:** Individual comparisons by virus and day post inoculation (DPI) of mosquito-sampled log-titer to syringe-sampled log-titer using the paired t-test and associated 95% CI.

Virus	DPI	Mean difference in log-titer	95% CI	p-value
HJV	1	0.30	−0.04–0.62	0.08
	2	−1.56	−2.75–−0.37	0.02
	3	−1.46	−3.67–0.76	0.11
	4	no observations	–	–
WNV	1	−0.80	−2.25–0.65	0.23
	2	−0.19	−0.65–0.28	0.37
	3	−0.15	−0.84–0.53	0.61
	4	−1.02	−2.33–0.30	0.11

### Precision of Mosquito Blood Meal Titers

There were 38 instances where multiple mosquitoes fed on the same viremic sparrow (HJV or WNV) and each mosquito was analyzed separately. When sparrow virus titers were >5.0 log_10_ pfu/mL serum, mosquito titers varied on average by 0.44 log pfu/mL serum from each other (range 0–1.5 log difference, n = 22). When sparrow titers were <5.0 pfu/mL serum, mosquitoes fed on the same animal had titers that varied on average by 2.7 log pfu/mL serum (range 0.2–4.7 log difference, n = 16).

### Viremia Determination in House Sparrows

**Figure 2:**
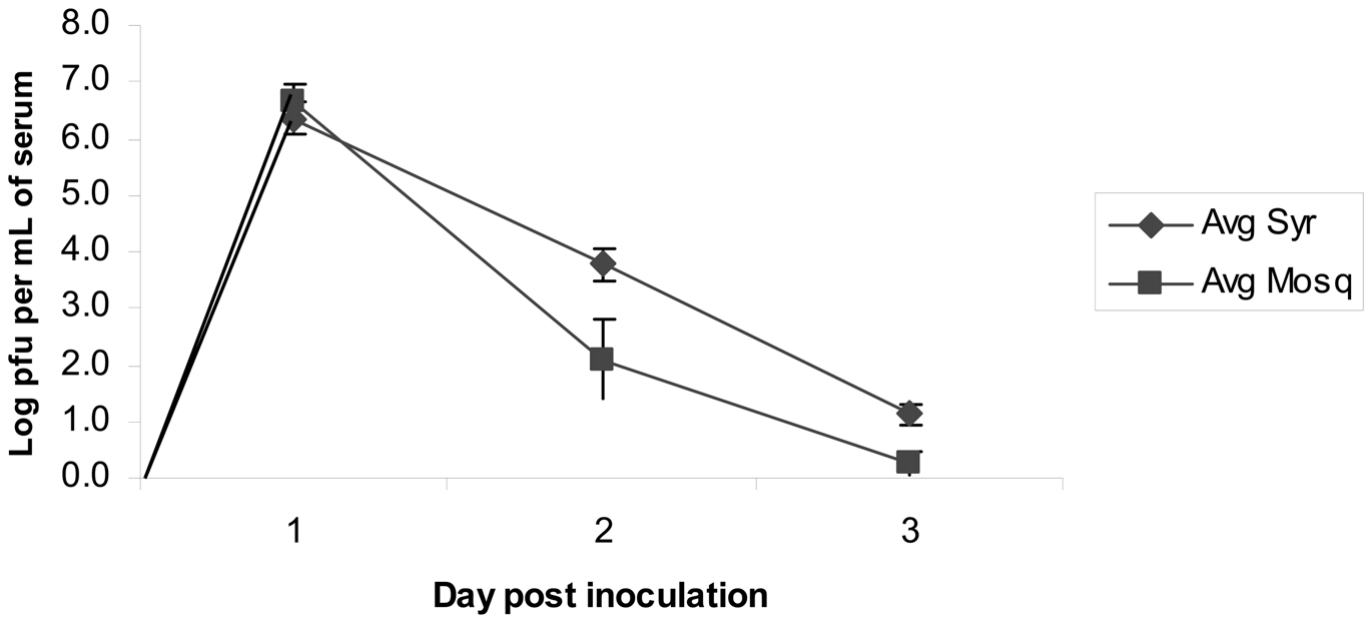
Mean daily titers (+/− SE) of HJV in house sparrows determined by syringe-drawn blood or individual mosquito blood meals.

**Figure 3:**
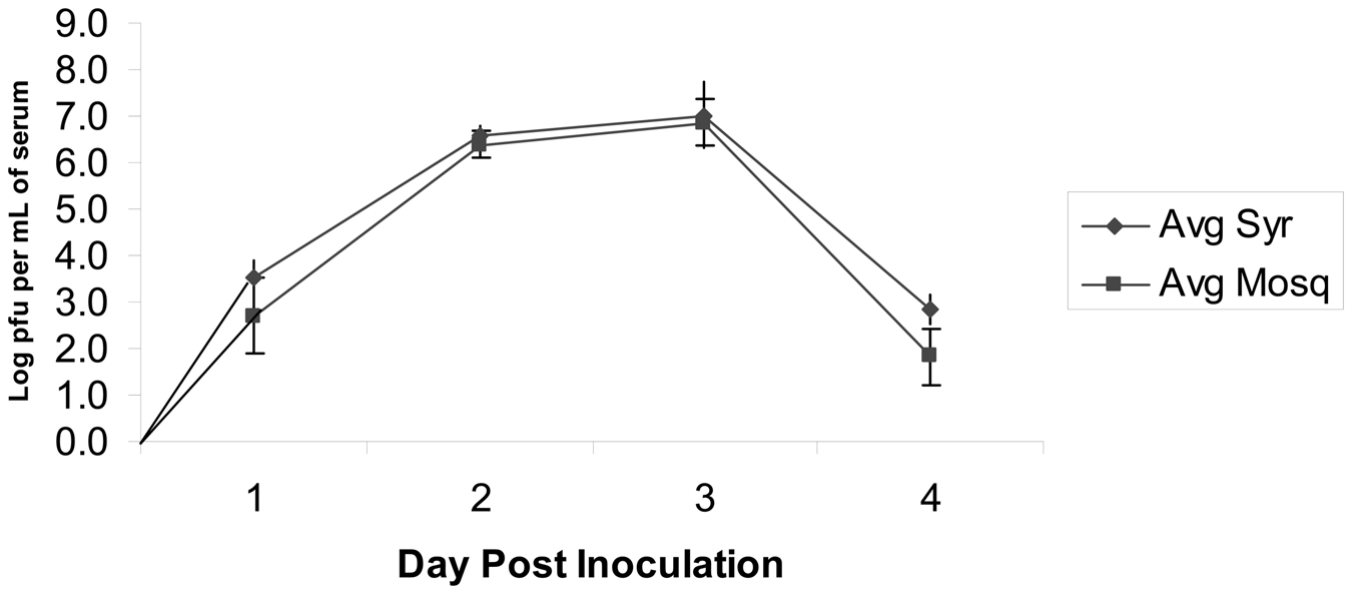
Mean daily titers (+/− SE) of WNV in house sparrows determined by syringe-drawn blood or individual mosquito blood meals.

Viremia profiles (mean daily viremia) were generated for house sparrow infected with HJV (Fig. 2) or WNV (Fig. 3). Comparison of the differences between mean log-titers derived by mosquito versus syringe at each dpi using the paired t-test adjusted for multiple comparisons within virus yielded no statistically significant differences for either WNV or HJV for any dpi. There were no observations for HJV at 4 dpi for comparison. We fit a linear mixed effects model for log-titer as a function of dpi for the HJV group, and a quadratic for log-titer as a function of dpi for the WNV group. The model for HJV indicated statistically significantly different slopes for the mosquito (−3.1; 95% CI −3.6, −2.6) and syringe collection methods (−2.0; 95% CI −2.6, −1.4). The quadratic model for WNV indicated no statistically significant difference in regression parameters by collection method. As noted in the methods section, we consider these modeling results as generally capturing the trends in these viremia profiles over this timeframe, but caution that we do not interpret these functional (i.e., linear and quadratic) relationships as definitive viremia profiles, but rather descriptive of trends.

### Test for Virus Adsorption to Mosquito Tissues

Mean virus titers were similar for WNV and HJV incubated with and without a homogenized *Ae*. *albopictus* mosquito (WNV 2.8 log_10_ pfu/mL vs. 2.9 log_10_ pfu/mL, HJV 3.0 log_10_ pfu/mL vs. 3.0 log_10_ pfu/mL, respectively). Therefore, any differences in virus titer between mosquito- and syringe-derived blood samples (i.e. day two titers for HJV in house sparrows) could not be explained by virus adsorption onto mosquito tissues during feeding or grinding.

## Discussion

We have evaluated a novel technique for viremia determination in vertebrates which uses mosquitoes in place of syringes for drawing blood. This technique can be applied to a variety of virus - vertebrate systems, and although demonstrated with a mosquito-borne flavivirus and alphavirus, is not limited to the study of arboviruses. Single engorged mosquitoes fed on viremic animals were successfully used to approximate mean daily virus titer and describe viremia profiles. Mosquito-derived blood also informed the day of maximum viremia, and the duration of detectable viremia. Importantly, fewer animals are required since individuals can be repeatedly sampled on consecutive days and blood samples can be obtained in a less invasive manner.

Mean daily titers determined from mosquito-drawn blood were usually slightly lower than titers determined by syringe (Figs. 2, 3). Although titer comparisons were not significantly different (p>0.05), this trend was observed for sparrows infected with HJV and WNV, as well as in chicks and hamsters. There are several potential explanations for this observation. First, limitations imposed by mosquito blood meal size lowered the probability that mosquitoes would imbibe virus particles when feeding on animals with low levels of circulating virus. This obstacle was overcome by feeding many mosquitoes (10–15 mosquitoes per animal) in order to detect virus in one or a few blood meals. Secondly, it is possible that some virus particles became sequestered to mosquito tissues either during feeding or the tissue grinding process and therefore were not present in the supernatant for analysis. This is unlikely, since mosquitoes were frozen almost immediately after they fed to repletion, and virus titers were similar in samples incubated with and without a homogenized mosquito. Thirdly, determining virus titer from mosquito blood meals is dependent upon having a reliable estimate of mosquito blood meal size. Because mosquito size, and hence blood meal volume, is highly variable, an unbiased estimate was difficult to obtain. We did not standardize mosquito size through tightly controlled rearing conditions in order to simplify the technique for use with mosquitoes pulled directly from maintained colony cages. Based on reports in the literature we assumed an average blood meal volume of 2 µl (1 µl serum)[15]–[17]; however this may have been high or low for some mosquitoes, thus skewing the estimated titer. A more accurate estimate of blood meal volume was attempted by weighing mosquitoes before and after feeding, however this was unsuccessful. Inherent variability in the plaque assay technique is also caused by estimating titer from serially diluted samples.

In looking at variability among virus titers in mosquito blood meals from the same sparrow, most mosquitoes that fed on a sparrow with a titer >5.0 log pfu/mL serum had blood meal titers within 0.5 log of each other. However in one case a few mosquitoes had very different titers from the rest. For example, 10 mosquitoes fed on a WNV-infected sparrow with a syringe titer of 6.0 log_10_ pfu/mL serum. For reasons that remain unclear, seven mosquitoes had titers ranging from 6.5 to 6.9 log_10_ pfu/mL serum, and three had titers of 9.7, 9.7 and 9.5 log_10_ pfu/mL serum. These results were re-confirmed by Vero cell plaque assay. Concentration of blood in the midgut post-feeding was considered, however mosquitoes were not given enough time to undergo diuresis prior to freezing, and no such droplets were ever seen at the bottom of the cup. Alternatively the location on the animal where those aberrant blood meals were obtained and uneven distribution of virus particles within the blood stream could possibly have played a role.

A viremia profile for house sparrows infected with HJV has not been previously reported. The sparrows reached a peak viremia within 24 hours post inoculation, after which viremia dropped off sharply (Fig. 2). This HJV profile is similar to that observed in two week old chicks and turkeys [1], [21]. Peak viremia exceeded the threshold of infection for *Culex pipiens* complex mosquitoes [22]–[23], therefore identifying the house sparrow as a competent amplifying host for HJV. Although 11% of wild-caught sparrows had HJV neutralizing antibodies (PRNT_50_) prior to experimentation, these antibodies were probably for a different alphavirus in the WEEV complex. Highlands J virus is only found in the eastern United States [24] but is in the WEE antigenic complex [25]. The majority (6/8) of those birds with alphavirus neutralizing antibody were hatch years, which would also indicate a recent exposure.

The viremia profile generated for house sparrows infected with WNV (NY99-4132) was different from that previously reported [11]. WNV viremia in house sparrows in this study peaked at three dpi at approximately 7.0 log_10_ pfu/mL serum, and dropped to below 3.0 log_10_ pfu/mL serum by four dpi. In contrast, Langevin et al. [13] demonstrated that WNV viremia in house sparrow lasted six days, with viremia peaking at five dpi at titers of approximately 10 log_10_ pfu/mL serum. The birds used in these two studies were captured in late summer – early fall in neighboring counties in northern Colorado and were all inoculated subcutaneously in the breast with the same strain of WNV. Birds captured in 2004 were inoculated with 10^3.2^ pfu/mL serum and 2007 birds were inoculated with 10^2.6^ pfu/mL serum. Attenuation in duration and/or magnitude of WNV viremia could be due to slight differences in virus inocula, differences in the way mean daily titer was calculated (mean of log values vs. log of mean values), differences in age and sex of the birds, or possible genetic adaptation of birds to consistently high levels of WNV transmission in Weld and Larimer Counties between 2003 and 2007 [26]–[28]. Other studies that generated WNV viremia profiles in Colorado populations of house sparrow several years after WNV introduction observed similarly lower peak titers for house sparrow [29].

## Conclusion

We have developed a novel technique for arbovirus viremia determination in small vertebrates. By testing the blood meals of single engorged mosquitoes fed on viremic animals, we have closely approximated the viremia profile, duration of detectable viremia, and day of peak viremia in chicks, hamsters, and sparrows infected with HJV and WNV. Additionally, we have applied the technique to generate a novel HJV viremia profile for house sparrow, and an updated WNV viremia profile in a Colorado population of house sparrows. While we have demonstrated the proof of concept by testing mosquitoes individually for comparison against syringe titer, future work should seek to compare the precision of pooling engorged mosquitoes fed on a viremic host against testing them individually for approximating virus titer. Additionally, the minimum number of mosquitoes necessary to obtain an accurate estimate of viremia will vary by titer, and has yet to be modeled. Still, this technique creates the opportunity to investigate the competence of other small vertebrates such as reptiles, rodents, and nestling birds as arbovirus amplifying hosts, which until this point has been impossible due limitations in serial blood sampling capabilities.
